# Late gadolinium enhancement cardiovascular magnetic resonance predicts clinical worsening in patients with pulmonary hypertension

**DOI:** 10.1186/1532-429X-14-11

**Published:** 2012-02-01

**Authors:** Benjamin H Freed, Mardi Gomberg-Maitland, Sonal Chandra, Victor Mor-Avi, Stuart Rich, Stephen L Archer, E Bruce Jamison, Roberto M Lang, Amit R Patel

**Affiliations:** 1 Section of Cardiology, Department of Medicine, University of Chicago Medical Center, Chicago, Illinois, USA; 2 Department of Radiology, University of Chicago Medical Center

## Abstract

**Background:**

Late gadolinium enhancement (LGE) occurs at the right ventricular (RV) insertion point (RVIP) in patients with pulmonary hypertension (PH) and has been shown to correlate with cardiovascular magnetic resonance (CMR) derived RV indices. However, the prognostic role of RVIP-LGE and other CMR-derived parameters of RV function are not well established. Our aim was to evaluate the predictive value of contrast-enhanced CMR in patients with PH.

**Methods:**

RV size, ejection fraction (RVEF), and the presence of RVIP-LGE were determined in 58 patients with PH referred for CMR. All patients underwent right heart catheterization, exercise testing, and N-terminal pro-brain natriuretic peptide (NT-proBNP) evaluation; results of which were included in the final analysis if performed within 4 months of the CMR study. Patients were followed for the primary endpoint of time to clinical worsening (death, decompensated right ventricular heart failure, initiation of prostacyclin, or lung transplantation).

**Results:**

Overall, 40/58 (69%) of patients had RVIP-LGE. Patients with RVIP- LGE had larger right ventricular volume index, lower RVEF, and higher mean pulmonary artery pressure (mPAP), all p < 0.05. During the follow-up period of 10.2 ± 6.3 months, 19 patients reached the primary endpoint. In a univariate analysis, RVIP-LGE was a predictor for adverse outcomes (p = 0.026). In a multivariate analysis, CMR-derived RVEF was an independent predictor of clinical worsening (p = 0.036) along with well-established prognostic parameters such as exercise capacity (p = 0.010) and mPAP (p = 0.001).

**Conclusions:**

The presence of RVIP-LGE in patients with PH is a marker for more advanced disease and poor prognosis. In addition, this study reveals for the first time that CMR-derived RVEF is an independent non-invasive imaging predictor of adverse outcomes in this patient population.

## Background

Significant advances in our understanding of the pathophysiology of pulmonary hypertension (PH) have led to several therapies that have improved quality of life and decreased mortality. Indeed, in this decade, 1-year survival rate is 85% versus 68% in the 1980s [1]. Despite this relative improvement in short-term survival, the prognosis of patients with PH remains poor [2]. Clinical management of these patients is driven, in part, by the ability to predict survival, but recent efforts to predict 1-year survival in patients with pulmonary arterial hypertension have not incorporated multiple non-invasive parameters such as right ventricular (RV) size and function [3].

Due to its relatively high intra- and inter-observer reproducibility, many have advocated the use of cardiovascular magnetic resonance (CMR) over echocardiography as a non-invasive way to predict outcomes and assess the effects of medical therapy on RV function over time [4-6]. Recently, several studies have highlighted the potential utility of CMR in patients with PH after discovering the presence of late gadolinium enhancement (LGE) in the right ventricular insertion point (RVIP) of the interventricular septum in the majority of these patients [7-11]. These studies suggested a significant inverse correlation between the degree of RVIP-LGE and right ventricular ejection fraction (RVEF) and hemodynamics.

While previous publications focused on the existence of RVIP-LGE in patients with PH and its association with multiple indices of RV failure, our study sought to investigate the potential role of RVIP-LGE and other CMR-derived parameters of RV function as non-invasive predictors of death, decompensated RV heart failure, initiation of prostacyclin or lung transplantation. Specifically, we hypothesized that: 1) the presence of RVIP-LGE significantly correlates with both the hemodynamic parameters of PH and associated RV findings, and 2) RV function, including RVIP-LGE, can be used to predict time to clinical worsening in patients with PH.

## Methods

### Study population and design

We evaluated 62 consecutive patients with PH referred for CMR as part of their clinical assessment between January 2009 and July 2010. Patients were excluded if they had an implantable cardioverter defibrillator or pacemaker, were claustrophobic, other contra-indication to CMR, or had a GFR of less than 30 ml/min/1.73 m^2^. All patients underwent right heart catheterization, exercise testing and measurement of N-terminal pro brain natriuretic peptide (NT-proBNP), but the results of these tests were included in the final analysis only if they were performed within 4 months of their CMR evaluation. Each patient underwent a thorough clinical evaluation by one of two physicians with expertise in PH (MG and SR), in which World Health Organization (WHO) functional status [12] was assessed and medications documented. The etiology of PH was recorded and classified according to the WHO schema [13]. Patient characteristics are summarized in Table 1* and *Table 2

**Table 1:** Clinical characteristics of patient population

	All patients (n = 58)
	
Demographics	
Age (years)	53 ± 14

Women, n(%)	43 (74%)

**Time of PH Diagnosis**	

Prior to CMR study	38 (66%)

After CMR study	20 (34%)

**WHO Categorizations**	

WHO Group I	44 (76%)

Idiopathic PAH	24

Associated PAH	20

WHO Group II	8 (14%)

WHO Group III	1 (1.7%)

WHO Group IV	2 (3.4%)

WHO Group V	3 (5.2%)

**Medications at time of CMR**	

Prostacyclin analogs ± other* PH medications	18 (31%)

Other* PH medications only	18 (31%)

No PH medications	22 (38%)

*Other medications include endothelin receptor antagonists, calcium channel blockers, phosphodiesterase inhibitors, and multikinase inhibitors (sorafenib)

**Table 2:** Comparison between diagnostic characteristics of patients with and without late gadolinium enhancement (LGE)

CMR	All patients (n = 58)	Patients with LGE (n = 40)	Patients without LGE (n = 18)	p value
Right ventricular end-diastolic volume index (ml/m2)	128 ± 57	137 ± 55	101 ± 55	0.03

Right ventricular end-systolic volume index (ml/m2)	84 ± 56	94 ± 53	57 ± 55	0.03

Right ventricular ejection fraction (%)	38 ± 15	35 ± 13	48 ± 14	< 0.01

Right ventricular mass index (g/m2)	27 ± 13	31 ± 13	19 ± 12	< 0.01

Right ventricular stroke volume (ml)	81 ± 26	81 ± 27	80 ± 22	0.98

Right atrial volume (ml)	133 ± 59	139 ± 59	112 ± 49	0.10

Right ventricular stroke work (mmHg × ml)	3776 ± 1766	3410 ± 2192	1934 ± 1581	0.02

Left ventricular end-diastolic volume index (ml/m2)	72 ± 27	72 ± 30	76 ± 21	0.51

Left ventricular end-systolic volume index (ml/m2)	32 ± 21	33 ± 22	33 ± 21	0.94

Left ventricular ejection fraction (%)	57 ± 10	56 ± 9.5	59 ± 11	0.34

Left ventricular mass index (g/m2)	41 ± 15	43 ± 17	37 ± 12	0.11

Left atrial volume (ml)	70 ± 33	65 ± 32	83 ± 33	0.06

**Naughton-Balke Exercise Treadmill Test**	All patients* (n = 42)	**Patients with LGE (n = 30)**	**Patients without LGE (n = 12)**	**p value**

Metabolic equivalents	5.6 ± 2.3	5.3 ± 2.3	6.5 ± 2.4	0.15

**Right Heart Catheterization**	All patients* (n = 35)	**Patients with LGE (n = 27)**	**Patients without LGE (n = 8)**	**p value**

Mean right atrial pressure (mmHg)	9.8 ± 5.0	10 ± 5.2	7 ± 3.0	0.08

Mean pulmonary artery pressure (mmHg)	49 ± 16	52 ± 16	35 ± 5.8	< 0.01

Pulmonary capillary wedge pressure (mmHg)	12 ± 5.4	12 ± 6.0	13 ± 3.1	0.68

Cardiac index (L/min/m2)	2.6 ± 0.99	2.4 ± 0.85	3.4 ± 1.3	0.08

Pulmonary vascular resistance (Wood Units)	9.5 ± 5.3	10 ± 5.7	5.2 ± 1.1	< 0.01

Mixed venous oxygen saturation (%)	62 ± 15	63 ± 11	66 ± 10	0.52

**N-Terminal Pro Brain Natriuretic Peptide**	All patients* (n = 43)	**Patients with LGE (n = 30)**	**Patients without LGE (n = 13)**	**p value**

NT-proBNP level (pg/mL)	2100 ± 4862	2334 ± 5316	705 ± 1330	0.13

* Only hemodynamics, METS, and NT-proBNP testing within 4 months of the CMR study were included in analysis.

Medical records were reviewed for the primary endpoint of time to clinical worsening. Time to clinical worsening includes 1) all cause mortality 2) hospitalization due to clinically decompensated right ventricular heart failure requiring IV therapy 3) initiation of prostacyclin or 4) lung transplantation. All deaths were confirmed by the social security death index. Whenever possible, the causes for hospitalization and death were recorded. The study was approved by the Institutional Review Board. While patients were prospectively tracked from the time of their CMR study to the primary end point or completion of study, hemodynamic data, functional analysis and NT-proBNP were retrospectively obtained in order to include a reasonably comprehensive, clinically relevant dataset in as many patients with PH as possible.

### CMR

CMR images were acquired on a 1.5-T scanner (Achieva, Philips, Best, Netherlands). Retrospectively gated cine images were obtained using the steady-state free precession (SSFP) sequence (TR 2.9 ms, TE 1.5 ms, flip angle 60°, and temporal resolution ~40 ms). Standard long-axis views were obtained, including four-chamber, two-chamber, and three-chamber images. In addition, one series of short axis slices that included both right and left ventricles from base to apex were acquired and a series of axial cines were acquired in order to determine atrial volumes. LGE images of the short and long axis views were obtained 10 minutes after infusion of gadolinium-diethylene triamine-pentaacetic acid (Gd-DTPA 0.2 mmol/kg) using T1-weighted gradient echo pulse sequence with a phase sensitive inversion recovery reconstruction (TR 4.5 ms, TE 2.2 ms, TI 250-300 ms, flip angle 30°, flip angle 5°, voxel size 2 × 2 × 10 mm, SENSE factor 2). An inversion time between 250 and 300 ms generally achieved successful nulling of the myocardium. During image acquisition, suspected partial volume and other artifacts seen on the LGE images were accounted for by either directly comparing the LGE image with an SSFP cine image taken at the same slice position or by swapping phase-encoding and frequency-encoding directions. If the existence of LGE was still in doubt, a second imaging plane was prescribed directly through the area of interest for further clarification.

### CMR image analysis

Images were analyzed using commercial software (Philips ViewForum, Best, Netherlands). Short axis slices were used to calculate left and right ventricular end-diastolic (first cine phase of the R wave triggered acquisition) and end-systolic (image phase with the smallest ventricular cavity area in the majority of slices) volumes, masses, and ejection fractions by the Simpson method of disks [14]. Left and right atrial volumes were calculated using the ventricular end-systolic frame from the axial cines. All volumes and masses were indexed for body surface area. The interventricular septum was considered part of the left ventricle for left ventricular mass calculation purposes. LGE of the myocardium was visually assessed by a CMR expert blinded to hemodynamic, functional, and laboratory data. LGE was considered to be present if the signal intensity in the myocardium at the RVIP was greater than or equal to that seen in the blood pool, present in 2 consecutive slices, and clearly present within the myocardium when compared against a matching SSFP cine image.

### Right heart catheterization

Although all patients had a right heart catheterization to initially diagnose PH, a subgroup of them underwent right heart catheterization (using a Swan-Ganz catheter) within 4 months their CMR study. Hemodynamics and mixed venous oxygen saturation were obtained. Cardiac output and index were calculated using the thermodilution technique. Pulmonary vascular resistance and right ventricular stroke work were recorded by standard formula.

### N-terminal pro-brain natriuretic peptide

Although a majority had an NT-proBNP drawn at some point during their PH evaluation, NT-proBNP was measured in a subgroup of patients using a commercially available assay (Roche, Indianapolis, IN) within 4 months of their CMR study.

### Exercise treadmill test

A subgroup of patients underwent exercise treadmill testing performed using the Naughton-Balke protocol [15], wherein treadmill time (in seconds) was converted to exercise metabolic equivalents (METs) as described previously [16]. Tests were interpreted using previously published sex-specific nomograms for metabolic equivalents [17,18]. Although a majority of patients had an exercise test performed at some point during their PH evaluation, only treadmill tests performed within 4 months of the CMR study were included in the final analysis.

### Statistical analysis

Categorical variables were expressed as percentages and continuous variables as mean ± standard deviation. We used an independent sample (unpaired) Student *t*-test (equal variances not assumed) to compare the means of normally distributed continuous variables in those patients with and without RVIP-LGE. The Mann-Whitney *U *test was used to compare the means for non-normally distributed variables. Categorical variables were compared using Fisher Exact or Chi-Square test. P values < 0.05 were considered statistically significant. In 26 randomly selected patients, interobserver reproducibility between the primary reader and an additional investigator was assessed by means of percent agreement and Cohen's kappa (κ) statistic. Cox proportional hazard analysis was performed to assess univariate and multivariate predictors of clinical worsening. Hazard ratios (HR) and 95% confidence intervals (CI) were calculated. Multivariable analysis was performed using six variables that were the most statistically significant in the univariate analysis and satisfied the linearity and proportional hazard assumptions for linear regression. These variables include left ventricular end-diastolic volume index, RVEF, mean pulmonary artery pressure (mPAP), METs, NT-proBNP, and RVIP-LGE. The discriminatory capacity of the independent predictors of outcome was investigated using non-parametric estimates of the area under the receiver operating characteristic (ROC) curves. Survival curves for RVIP-LGE and each of the multivariate predictors of outcome were constructed with the Kaplan-Meier method and were compared by means of the log-rank test. The threshold values for each multivariate predictor of outcome were selected based on highest specificity and sensitivity as generated by the previously mentioned ROC curves. All statistical analyses were performed using Prism (GraphPad Software, San Diego, California) or Stata (version 11, StataCorp LP, College Station, Texas). MIX 2.0 software was used to prepare the forest plot graphs [19].

## Results

### Patient characteristics

Of the 62 patients, RVIP-LGE was indeterminate in 3 patients due to technical issues resulting in poor signal to noise ratio and excessive motion artifact in 1 patient. These 4 patients were not included in the final analysis. A total of 35 patients underwent right heart catheterization, 43 patients underwent NT-proBNP evaluation, and 42 patients underwent exercise stress testing within 4 months of their CMR study. During the mean follow-up period of 10.2 ± 6.3 months, 19/58 (33%) patients reached the primary endpoint of time to clinical worsening: 6 patients died, 11 were hospitalized for decompensated right ventricular heart failure, and 2 patients were hospitalized for initiation of prostacyclin analog. Of the patients who reached the primary endpoint, RVIP-LGE was present in 18/19 (95%). The patient without RVIP-LGE had sarcoidosis and was hospitalized for PH exacerbation.

### RVIP-LGE and association with invasive and non-invasive variables

**Figure 1:**
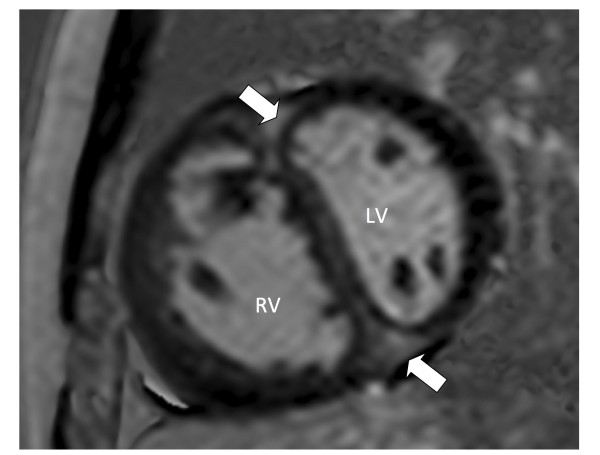
**Late gadolinium enhancement of right ventricular insertion point**. This figure depicts a short axis, late gadolinium enhanced, phase-sensitive CMR image of the left and right ventricle. The white block arrows indicate areas of LGE located in both the anterior and inferior RVIP. The mPAP for this patient at rest during right heart catheterization was 62 mmHg. LV = left ventricle; RV = right ventricle.

Overall, RVIP-LGE was present in 40/58 (69%) of patients (Figure 1). In comparing CMR variables (Table 2), patients with RVIP-LGE had significantly larger right ventricular volumes, lower RVEF, increased right ventricular mass index, and higher right ventricular stroke work. Patients with RVIP-LGE who underwent right heart catheterization had significantly higher mPAP and higher pulmonary vascular resistance. Right atrial pressure and cardiac index were not statistically significant; however, both variables trended in that direction. In those patients who underwent exercise or NT-proBNP testing, there was no statistical difference in METs or NT-proBNP between the patients with RVIP-LGE and those without.

### Reproducibility of RVIP-LGE

There was good interobserver agreement in identifying the existence of RVIP-LGE between the primary reader and an additional investigator (88%, κ = 0.72).

### Univariate and multivariate predictors of mortality

**Figure 2:**
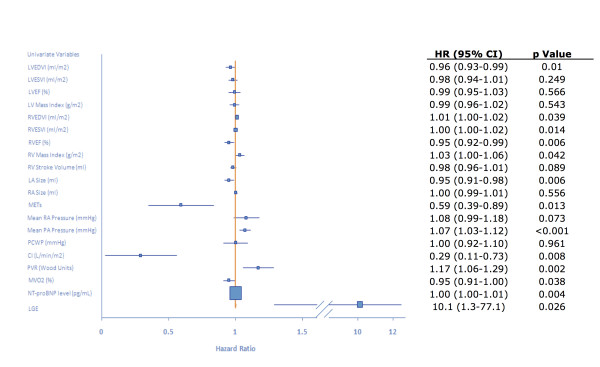
**Univariate analysis of multiple parameters for right ventricular function**. Forest plot of univariate proportional hazards modeling including hazard ratios, 95% confidence intervals and p-values for parameters obtained from CMR, functional testing, NT-proBNP, and hemodynamics. The presence of RVIP-LGE was statistically significant for predicting time to clinical worsening. LVEDVI = left ventricular end-diastolic volume index; LVESVI = left ventricular end-systolic volume index; LVEF = left ventricular ejection fraction; RVEDVI = right ventricular end-diastolic volume index; RVESVI = right ventricular end-systolic volume index; LA size = left atrial volume; RA size = right atrial volume; Mean RA Pressure = mean right atrial pressure; PCWP = pulmonary capillary wedge pressure; CI = cardiac index; PVR = pulmonary vascular resistance; MVO2 = mixed venous oxygen saturation.

The HR, CI, and statistical significance for all invasive and non-invasive variables are reported in Figure 2. The presence of RVIP-LGE was statistically significant for predicting time to clinical worsening. Other CMR univariate predictors of the primary outcome, in order of statistical significance, included: RVEF, left atrial volume, left ventricular end-diastolic volume index, right ventricular end-systolic volume index, right ventricular end-diastolic volume index, and right ventricular mass index. Hemodynamic univariate predictors of time to clinical worsening, in order of statistical significance, included: mPAP, pulmonary vascular resistance, cardiac index, and mixed venous oxygen saturation. NT-proBNP and METs were also significant for predicting the primary outcome. The multivariate analysis demonstrated that, after inclusion of six select statistically significant univariate predictors of time to clinical worsening, only mPAP (HR 1.14; 95% CI 1.05-1.23; p = 0.001), METs (HR 0.46; 95% CI 0.25-0.83, p = 0.01), and RVEF (HR 0.91; 95% CI 0.83-0.99, p = 0.036) remained statistically significant.

### Kaplan-Meier survival analysis

**Figure 3:**
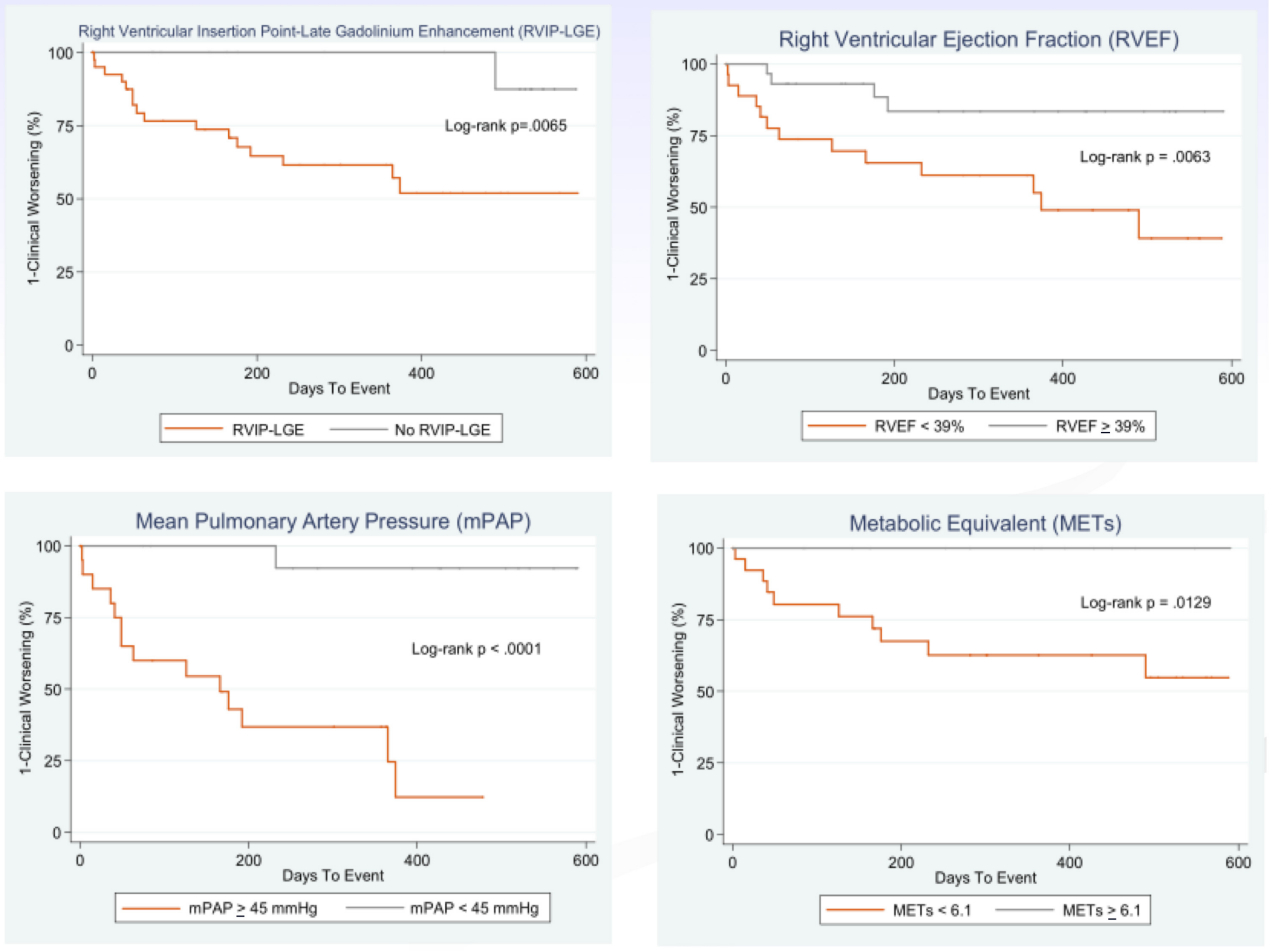
**Time to clinical worsening for patients with pulmonary hypertension**. Kaplan-Meier curves demonstrated time to clinical worsening for (A) patients with and without the presence of RVIP-LGE, (B) patients with RVEF ≥ 39% and < 39%, (C) patients with mPAP ≥ 45 mmHg and < 45 mmHg, and (D) patients with METs ≥ 6.1 and < 6.1.

Kaplan-Meier survival curves generated for RVIP-LGE and the three independent predictors of time to clinical worsening are shown in Figure 3. Patients with RVIP-LGE were statistically more likely to reach the primary endpoint than those without the presence of RVIP-LGE (log-rank test, p = 0.0065). Patients with mPAP ≥ 45 mmHg (log-rank test, p = 0.0001), RVEF less than 39% (log-rank test, p = 0.0063), or METs ≤ 6.1 (log-rank test, p = 0.0129) also had a significantly worse prognosis.

## Discussion

In this study, we found that in patients with PH, not only is RVIP-LGE associated with RV dilation and hypertrophy, reduced RVEF, and more extensive hemodynamic abnormalities, but its presence is also a univariate predictor of time to clinical worsening. Although a few previous publications have discussed the existence and extent of RVIP-LGE in patients with PH and its inverse correlation with RV function, we have demonstrated in this study that it is also a non-invasive marker of adverse outcomes. In a multivariate analysis, although RVIP-LGE is no longer statistically significant, we show that CMR-derived RVEF is an independent predictor of time to clinical worsening along with known important clinical parameters of PH such as mPAP and METs.

### Previous publications

In 2005, Blyth et al. reported that 23/25 (92%) of their patients with PH had RVIP-LGE and that the extent of LGE was highly correlated with RV volume, RV mass, mPAP, and inversely correlated with RVEF [7]. A second study by Sanz et al. found that 41/42 (97%) patients with PH had evidence of RVIP-LGE while only 3/13 (23%) of patients without PH had RVIP-LGE [9]. In addition, similar to Blyth et al., the extent of RVIP-LGE correlated moderately well with multiple CMR and hemodynamic parameters of RV dysfunction. In a multivariate analysis, only systolic PAP predicted the presence of RVIP-LGE. Another study with 15 patients by McCann et al. reported the presence of RVIP-LGE in 100% of their PH patients and also demonstrated a positive correlation between extent of LGE and other CMR findings of RV dysfunction [8]. However, unlike Blythe et al. and Sanz et al., they did not find a significant correlation between the extent of LGE and hemodynamic parameters of PH, despite the fact that their cohort of patients had a higher overall mPAP when compared to the study by Blyth et al. (54 ± 16 mmHg vs. 43 ± 12 mmHg) and Sanz et al. (54 ± 16 mmHg vs. 44 ± 12). The most recent study examining RVIP-LGE in patients with PH showed RVIP-LGE in only 13/20 patients and the only significant correlation was found between the extent of LGE and the duration of disease and not with RV size, function, or hemodynamics [10]. Our study is in agreement with the findings by Blyth et al. and Sanz et al. but builds on the relatively small amount of data available on this topic by including more patients and additionally following them over time.

### The prevalence of RVIP-LGE in patients with PH

The prevalence of RVIP-LGE in PH patients is not clearly defined; in fact, some previous publications have reported it as a nearly universal finding [8]. In our study population, approximately two-thirds of patients had RVIP-LGE. The 18 patients without RVIP-LGE had higher RVEF and lower mPAP suggesting less severe disease. Therefore, unlike the results of previous studies, the wider variety of PH etiologies (as reflected by inclusion of patients from all 5 WHO groups) and greater spectrum of disease severity in our patient cohort showed that RVIP-LGE is not necessarily a ubiquitous marker for PH but rather is more directly related to reduced RV function and, hence, disease severity.

### Non-invasive predictors of outcome

Since the presence of RVIP-LGE is highly associated with reduced RV function, we followed all patients for an average of 10 months and found that patients with RVIP-LGE were ten times more likely to reach the primary endpoint of clinical worsening than those patients without RVIP-LGE. While significantly predictive in a univariate analysis, the presence of RVIP-LGE was not an independent predictor of poor outcomes in a multivariable analysis likely due to its strong association with other variables included in the model. However, the data suggest that it is a strong marker for detecting more severe disease manifested by higher mPAP and lower RVEF and functions as a simple, binary, reproducible, and visible reflection of these independent predictors of clinical worsening. Furthermore, the absence of RVIP-LGE predicted 100% survival in 14 months whereas only half of the patients with evidence of RVIP-LGE were alive or free from hospitalization or prostacyclin initiation in the same time frame.

In our patient cohort, RVEF was the only independent prognostic variable obtained from the CMR examination. We also confirm previous reports [20] that mPAP and METs were independent predictors of poor prognosis. Our results are consistent with previous studies showing that RVEF is, indeed, a powerful predictor of adverse outcomes in patients with PH [21-23]. Kawut et al. showed, in a retrospective cohort of 84 patients with PAH, that radionuclide angiography-derived-RVEF is independently associated with mortality [23]. Obvious advantages of using CMR-derived RVEF over nuclear-derived RVEF to risk stratify PH patients are that no intravenous line is needed and there is no exposure to ionizing radiation. The thin free wall of the RV and the extensive trabeculation of the chamber can make quantitative assessment of the RVEF challenging. In addition, it requires some expertise to accurately define the most basal short-axis slice of the RV. Despite this, the reproducibility of CMR-derived RVEF is excellent and it is generally regarded as the reference standard for the measurement of RV size and function [6].

In the only other publication studying the prognostic significance of CMR variables in patients with PH, van Wolferen et al. performed CMR, right heart catheterization, and the six minute walk test at baseline and 1-year later in patients with idiopathic pulmonary arterial hypertension [21]. They found that stroke volume index, right ventricular end-diastolic volume index, and left ventricular end-diastolic volume index were independent predictors of mortality and treatment failure at baseline but RVEF was not. This discrepancy in results might be due to the fact that we included multiple etiologies of PH in our study or that the length of our follow-up was different. In addition, in their study, RVEF was calculated by dividing stoke volume (which was obtained using velocity-encoded imaging of the pulmonary artery) by RVEDV. This difference in methods for calculating RVEF likely explains the discrepancy in our results. Nevertheless, both studies confirm that CMR provides meaningful information, particularly about the right ventricle, that can help risk stratify patients so that they may receive appropriate management.

### Suspected etiology of RVIP-LGE

The cause of RVIP-LGE in patients with PH remains to be fully elucidated. Myocardial infarction is highly unlikely, given the atypical pattern and focal distribution of RVIP-LGE. Although several studies have shown reversible ischemia in the right ventricle, particularly in PH patients with elevated pulmonary pressures, none have shown fixed perfusion defects in the RVIP [24]. Myocardial fibrosis due to mechanical stress and strain of the RVIP with elevated RV pressures has previously been implicated [7]. However, others have suggested that RVIP-LGE may represent myocardial disarray rather than fibrosis [25]. Indeed, Bradlow et al. examined the heart of a patient with idiopathic pulmonary arterial hypertension at autopsy who had evidence of RVIP-LGE [26]. They found increased collagen and fat between fiber bundles (plexiform fibrosis) consistent with myocardial disarray, but no pathologic fibrosis. Disarray is common in the RVIP even in healthy patients but becomes exaggerated in the presence of RV hypertrophy and dilatation and, thus, allows for more contrast pooling. This may be the reason why then, in our study, RVIP-LGE was associated with larger right ventricular volume and mass but failed to add prognostic value over known independent markers for death and treatment failure. Another potential explanation for RVIP-LGE includes partial volume effect created by contrast trapped within the extensive trabeculation of the dilated RV cavity adjacent to the actual RVIP rather than within the actual myocardium itself.

### Limitations

One important limitation of our study is that only a subset of patients underwent right heart catheterization, NT-proBNP testing and/or functional testing within 4 months of their CMR exam. However, we felt it important to include these data (despite the above limitation) in our analysis because PH is a rare disorder with limited opportunities for study and that it would be important to define the prognostic value of CMR within the context of other clinically relevant variables. Another important limitation of our study is that we were unable to reliably quantify the amount RVIP-LGE due to partial volume effect, preventing us from accurately defining the borders of hyperenhancement in every patient. We believe, however, that there is significant clinical utility in being able to easily visualize the presence of RVIP-LGE and using it as a marker for detecting more severe disease and worse outcomes.

## Conclusion

Contrast-enhanced CMR may be a useful tool in the management of patients with PH. RVIP-LGE is a readily visible, non-invasive marker associated with more advanced PH, as reflected by its association with larger RV volumes and greater RV mass, reduced RVEF, and higher mPAP. Patients with RVIP-LGE are significantly more likely to worsen clinically than those without this marker. Additionally, CMR-derived RVEF is an independent predictor of adverse outcomes in patients with PH and its ability to risk stratify PH patients is similar to that of mPAP and METs. As new medications for PH are developed and patients with this disease are treated earlier in their disease course, contrast-enhanced CMR could potentially help in risk stratification so that appropriate therapy can be given in a timely manner.

## Competing interests

SLA: NIH-RO1-HL071115 and 1RC1HL099462-01, the American Heart Association, and the Roche Foundation for Anemia Research

MGM: Actelion, Gilead, Lilly/Icos, Pfizer, Novartis, and United Therapeutics have provided funding to the University of Chicago to support conduct of clinical trials. MGM has served as a consultant/participant on data safety monitoring board/steering committee for clinical trials for Actelion, Gilead, Medtronic, and Pfizer. MGM does not do any promotional speaking. MGM has a patent filed entitled, "Compositions and Methods for Treating Pulmonary Hypertension" for the use of sorafenib in pulmonary hypertension. WO/2007/087575

## Authors' contributions

BHF was involved in acquiring and interpreting the data. He was primarily responsible for drafting the manuscript. MGM, SR, and SLA conceived of the idea for the project and helped critically revise the manuscript for intellectual content. SC and EBJ acquired and interpreted the data. ARP and RML helped conceive of the idea for the project, analyze the data, and critically revise the manuscript for intellectual content. VM helped analyze the data and helped in drafting the manuscript. All authors gave final approval of this version to be published.
